# Editorial for the Special Issue “Advances in Molecular Microbiology, Genetics, and Bioinformatics of Multiple-Drug-Resistant Bacteria in Public Health”

**DOI:** 10.3390/genes17040366

**Published:** 2026-03-24

**Authors:** Pedro Panzenhagen, Carlos Adam Conte-Junior

**Affiliations:** 1 Graduate Program in Biochemistry (PPGBq), Institute of Chemistry (IQ), Federal University of Rio de Janeiro (UFRJ), Cidade Universitária, Rio de Janeiro 21941-909, RJ, Brazil; conte@iq.ufrj.br; 2 Center for Food Analysis (NAL), Technological Development Support Laboratory (LADETEC), Federal University of Rio de Janeiro (UFRJ), Cidade Universitária, Rio de Janeiro 21941-598, RJ, Brazil; 3 Laboratory of Advanced Analysis in Biochemistry and Molecular Biology (LAABBM), Department of Biochemistry, Federal University of Rio de Janeiro (UFRJ), Cidade Universitária, Rio de Janeiro 21941-909, RJ, Brazil; 4 Graduate Program in Veterinary Hygiene (PGHIGVET), Faculty of Veterinary Medicine, Fluminense Federal University (UFF), Niterói 24230-340, RJ, Brazil; 5 Graduate Program in Food Science, Institute of Chemistry, Federal University of Rio de Janeiro, Rio de Janeiro 21941-909, RJ, Brazil; 6 Graduate Program in Microbiology (PPGMicro), Department of General Microbiology, Institute of Microbiology Paulo de Góes (IMPG), Federal University of Rio de Janeiro, Rio de Janeiro 21941-971, RJ, Brazil; 7 Analytical and Molecular Laboratory Center (CLAn), Institute of Chemistry (IQ), Federal University of Rio de Janeiro (UFRJ), Cidade Universitária, Rio de Janeiro 21941-598, RJ, Brazil

Antimicrobial resistance (AMR) has emerged as one of the most significant global threats to public health, food safety, and environmental sustainability. The rapid emergence and dissemination of multidrug-resistant (MDR) bacteria compromise the effectiveness of available antimicrobial therapies and complicate the management of infectious diseases worldwide. Advances in molecular microbiology, genomics, and bioinformatics have significantly improved the ability to investigate bacterial evolution, resistance mechanisms, and pathogen dissemination at high resolution. These approaches enable the identification of resistance determinants, virulence factors, and mobile genetic elements that contribute to the adaptation and persistence of bacterial pathogens in diverse ecological niches. This Special Issue brings together nine contributions that explore bacterial evolution, genomic diversity, resistance and virulence determinants, and potential strategies for combating MDR pathogens across clinical, environmental, and food-related contexts.

Several articles focus on the genomic characterization of bacterial pathogens associated with food production systems. Zhang et al. conducted whole-genome sequencing and comparative genomic analysis of goat-derived *Klebsiella oxytoca*, providing insights into the genomic features, resistance determinants, and potential virulence factors of this opportunistic pathogen. Their findings highlight the relevance of genomic surveillance in understanding zoonotic risks and the potential dissemination of antimicrobial resistance through livestock reservoirs [1]. Panzenhagen et al. investigated the global population structure of *Salmonella* Saintpaul using comparative genomic analyses. By examining genomic diversity and epidemiological patterns, the authors described the evolutionary dynamics and outbreak potential of this important foodborne pathogen. Their study provides valuable insights into the global dissemination of this serovar and highlights the role of genomic epidemiology in foodborne disease surveillance [2]. Another contribution explored the genomic characteristics of *Salmonella*. Using whole-genome sequencing and comparative genomic analysis, Nunes et al. identified distinct virulence gene profiles, antimicrobial resistance determinants, and plasmid content among strains of *Salmonella* Cerro and *Salmonella* Schwarzengrund isolated from food products in Brazil. These findings demonstrate the value of genomic approaches in characterizing foodborne pathogens and assessing their potential impact on public health [3].

Comparative genomic approaches are also applied to investigate the emergence of globally disseminated resistant lineages. Ahmed et al. analyzed the metabolic and genomic adaptations of fluoroquinolone-resistant *Salmonella* Kentucky ST198, a lineage associated with human infections worldwide. Their results revealed lineage-specific metabolic traits and enhanced respiratory activity that may contribute to the ecological success and global spread of this pathogen [4].

Clinical pathogens are also represented in this Special Issue. Cavka et al. investigated multidrug-resistant *Pseudomonas aeruginosa* isolates related to ventilator-associated pneumonia and ventilator-associated tracheobronchitis in an intensive care unit. Whole-genome sequencing revealed the presence of multiple resistance determinants, virulence genes, and high-risk sequence types, highlighting the complexity of antimicrobial resistance and virulence in hospital-associated infections [5].

In addition to pathogenic bacteria, some studies address the role of environmental and commensal microorganisms as reservoirs of antimicrobial resistance genes. Mikołajczuk-Szczyrba et al. examined lactic acid bacteria isolated from livestock environments and identified multiple resistance determinants through genomic analysis. The presence of plasmid-encoded resistance genes suggests that commensal bacteria may contribute to the horizontal dissemination of antimicrobial resistance across microbial communities [6]. Similarly, Zawiasa and Olejnik-Schmidt investigated antimicrobial resistance in *Listeria innocua* isolated from food products and processing environments. Although this species is generally considered non-pathogenic, the study revealed heterogeneous resistance profiles and the presence of resistance-associated genes, emphasizing the importance of monitoring non-pathogenic species as potential reservoirs of antimicrobial resistance [7].

Alternative antimicrobial strategies are also explored in this collection. Andrzejczuk et al. evaluated the antimicrobial and antibiofilm activity of *Arnica montana* extracts against *Acinetobacter baumannii*, an opportunistic pathogen known for its high levels of antibiotic resistance and ability to form persistent biofilms. The study demonstrated that plant-derived extracts can inhibit biofilm formation and downregulate genes associated with bacterial adhesion, suggesting potential applications for natural compounds in antimicrobial therapy [8].

Finally, Sánchez-Corona et al. explored the ecological relationship between heavy metal contamination and antimicrobial resistance. Their work highlights how heavy metal resistance genes frequently co-occur with antibiotic resistance determinants within mobile genetic elements, promoting co-selection and persistence of multidrug-resistant bacteria in contaminated environments. These findings reinforce the importance of considering environmental reservoirs in the global epidemiology of antimicrobial resistance [9].

Collectively, the articles presented in this collection demonstrate the importance of integrating molecular microbiology, genomic technologies, and bioinformatic tools to advance our understanding of antimicrobial resistance. These approaches provide essential insights into pathogen evolution, resistance dissemination, and potential strategies for combating MDR bacteria. We thank all authors, reviewers, editors and the editorial staff who contributed to this Special Issue and hope that this collection will serve as a valuable reference for researchers working in the fields of microbial genomics, antimicrobial resistance, and public health microbiology.
